# Treatment of Medial Tibial Stress Syndrome according to the Fascial Distortion Model: A Prospective Case Control Study

**DOI:** 10.1155/2014/790626

**Published:** 2014-10-14

**Authors:** Christoph Schulze, Susanne Finze, Rainer Bader, Andreas Lison

**Affiliations:** ^1^Department of Orthopaedics, University Medicine Rostock, Doberaner Street 142, 18057 Rostock, Germany; ^2^Bundeswehr Centre of Sports Medicine, Dr.-Rau-Allee 32, 48231 Warendorf, Germany

## Abstract

Medial tibial stress syndrome (MTSS) is a common problem among athletes and soldiers. There is no proven theory that could explain the pathophysiology of shin splints. The therapies described so far are time-consuming and involve a high risk of relapse. The method according to the fascial distortion model (FDM) addresses local changes in the area of the lower leg fascia. It is suited to reduce pain and functional impairments associated with this symptom complex by applying targeted manual techniques. 32 patients (male: 30; female: 2) participated in this study. Visual analogue scale (VAS) was used for the quantification of pain. Scores were also given to rate the maximum painless exercise tolerance of the patients. Subsequently treatment of the crural fascia was performed. Patients retested ability of running and jumping. Therapy was continued until full exercise tolerance or painlessness was reached. A significant reduction of the VAS pain score from 5.2 to 1.1 could be achieved (*P* < 0.001). The impairment of exercise tolerance could be reduced from 7 to 2 points (*P* < 0.001). The duration of treatment was 6.3 (SD: 4.3) days on average. The FDM therapy is a potential effective method for acute treatment of MTSS.

## 1. Introduction

In 1966 the term “shin splints” was defined by the American Medical Association in the “Standard Definition of Athletic Injuries.” At that time, the definition was as follows: “pain and discomfort in the leg from repetitive running on hard surfaces or forcible extensive use of flexors…” [1]. This definition has been called into question over the years. Symptoms usually occur during running or sometimes even during walking without having any trauma. Pain reduces in times without physical stress but relapses when the athletes do sports again [2]. Various theories on the cause of pain in the lower leg were elaborated. Some authors assumed elevated muscle compartment pressures in the lower leg [3]. However, no increased compartment pressures were determined in the lower legs. The hypothesis of periostitis [4] has not yet been corroborated by a histological correlate [5]. Beck and Moen et al. described the theory that it is caused by bony resorption that outpaces bone formation of the tibial cortex [5, 6]. Various names have been introduced for the complex of this kind of musculoskeletal disorder: medial tibial syndrome, medial tibial stress syndrome (MTSS) [4], shin soreness [7], anterior compartment syndrome [1], and posteromedial compartment syndrome [1]. The incidence among physically active soldiers is 7.9%, and among athletes it varies between 4% and 35% [8, 9]. Smoking habits and stamina have also been identified as influencing factors [8]. Flattening of the longitudinal arch of the foot, increased plantar flexion in the upper ankle joint, and a restriction of internal hip rotation are described as biomechanical factors contributing to the development of MTSS [10, 11]. An increased body mass index has an adverse effect on the duration of MTSS [12]. The diagnosis of soft tissue injury is made through clinical examination; other related conditions may be ruled out with imaging or other diagnostic testing [13].

“Gold standards” exist for stress fracture and exertional compartment syndrome. There is a lack of a “gold standard”—particularly for soft tissue injury [14]. Treatment options range from rest, local cooling, and application of local and systemic anti-inflammatory medicaments to physiotherapy with ultrasound, phonophoresis, and local friction. However, none of these methods has proved superior to the others [14, 15]. The shock wave therapy has also been described as a successful therapy option and helps to reduce the duration of treatment [9, 16]. If the lower extremity, in particular the feet, shows an abnormal position, insoles can be used to provide biomechanical arch support. Shock-absorbing insoles can help to ameliorate symptoms [13, 15]. The application of pneumatic splints to the lower leg has no effect on the outcome of soft tissue injuries but may be helpful in case of stress fracture [17]. Targeted strengthening of the muscles stabilising the plantar arch can also be pursued as a therapeutic approach. This may also include proprioceptive activities [5, 18].

The treatment method derived from the fascial distortion model is based on an anatomical model and was introduced by Typaldos [19]. During the presentation of symptoms, the patient's body language is observed to make a diagnosis, choosing one from the six diagnoses which the fascial distortion model comprises. These diagnoses describe the conception of how the fasciae are twisted in a specific body segment where they cause specific problems [20]. This is used as a basis for deriving a specific technique that addresses the fascia. Other authors postulated that the fascia plays an important role in pathophysiology of MTSS. Bouché and Johnson hypothesized that with contraction of the deep leg flexors tension would be imparted to the tibial fascial attachment at the medial tibial crest and that circumferential straps would dampen tension directed to the medial tibial crest [20]. Stickley et al. described the tibial attachments of the deep crural fascia and concluded deep crural fascia involvement in creating MTSS [21].

The treatment of fascial distortions is based on targeted manual techniques which are to be described as nonspecific. Therapeutic success was described for shoulder pain, in particular for the management of frozen shoulder [22, 23]. In such cases, improvements regarding function and pain sensation could be quickly achieved. In comparison with conventional physiotherapy, the FDM method seems to be clearly superior. The objective of the present case control study is to demonstrate the effectiveness of this therapy for MTSS.

## 2. Materials and Methods

### 2.1. Patients

A total of 32 patients (male : female = 30 : 2) participated in the study. The patients were soldiers who participated in sports courses at the Bundeswehr Sports School in Warendorf. The study was approved by the Ethics Committee of the University of Muenster (Ref. no. 2012-200-f-S). On average, the patients were 26.3 ± 4.1 years old, weighed 80.9 ± 10.4 kg, and were 1.79 ± 0.07 m tall. Their average BMI was 25.3 ± 2.6 kg/m^2^. The study included patients who were diagnosed with MTSS, because they showed the typical symptoms of pain following exercise as well as tenderness of the anterior or posterior medial and lateral anterior surfaces of the tibia [2, 23]. In some cases the symptoms were acute (19; present for 1 week), and in other cases they had already been present for a long time (6; present for 8.5 weeks in mean). In some patients the symptoms had been present for several years (7; 2.5 years in mean). Patients who also had other pain syndromes in the lower leg (e.g., achillodynia) and patients who were not available for a therapy period of at least 10 days were excluded from the study. No further technical examinations like X-ray have been done prior to treatment with FDM. All patients were observed by the same physician. The physician had special education in sports medicine and manual therapy.

### 2.2. Quantification of Complaints

The quantification of pain was based on the visual analogue scale (VAS). The patients rated the intensity of their pain on a scale with numerical values between 0 (no pain) and 10 (the strongest pain imaginable). Painless running for defined periods of time or courses was used to quantify exercise tolerance [11]. We had to adapt this to military requirements. For the quantification of exercise tolerance, a score was designed to determine the tolerance to running (speed and distance that can be run without pain) and jumping. The gradations are shown in Table 1.

### 2.3. Treatment Method according to the Fascial Distortion Model

The patients were asked to indicate the location of pain. The diagnosis was derived from the body language and the description of the patient, using the fascial distortion model according to Typaldos [19]. The MTSS is mainly continuum distortions (palpable local transformation disorder of the fascia in the transition area from bone to body fascia) and trigger bands (the body fascia is twisted along the limb in longitudinal direction). Subsequent to diagnosis, the therapy was initiated using targeted manual techniques. To get an impression, techniques are presented in internet videos [24]. Mainly strong local pressure was applied on the painful points using the fingertip of the thumb. The pressure was reduced when the patient reported that there was no pain left. Alternatively the fingertip was swept along the tibia with strong pressure. These procedures were repeated until no further pain was reported in this session and the patient feels comfortable. No other forms of therapy were implemented.

### 2.4. Procedure of the Study

Subsequent to diagnosis, the patients were informed about the therapy according to the fascial distortion model. The patients who had decided to undergo this therapy were treated after signing the declaration of consent. Prior to that, they were asked to indicate their current exercise tolerance according to the scoring matrix. Here they were asked what distance they were able to run and if running or even walking was impossible. Further they were asked at what speed they were able to run. The third dimension was the ability to jump. They had to decide if jumping was possible or not and if there was slight or severe pain. High levels were addicted to high limitation. The value of each dimension is shown in Table 1. The pain levels were recorded according to the visual analogue scale (VAS). Here the patients have to choose a number between 0 and 10 where 0 means no pain and 10 means the highest imaginable pain level. After treatment, the patients (participants in a sports course) were not allowed to perform any sporting activities involving running or jumping on the same day. The following morning, the therapeutic success was evaluated by the patients. They were asked to assess their exercise tolerance and pain sensation during the course and to quantify these parameters again by using the tolerance scores and the VAS. When a patient was free of symptoms, the therapy was terminated; when symptoms persisted, the therapy was continued. The therapy was terminated upon achievement of absence of pain and full exercise tolerance, at the end of the course or upon request of the patient.

### 2.5. Statistical Evaluation

The statistical description included the determination of maximum, minimum, mean value, and standard deviation. The statistical evaluation and significance test were performed by using the Wilcoxon test. All *P* values were two-tailed and a *P* value < 0.05 was considered significant. Data were stored and analysed using SPSS 15.0 software (SPSS Inc., Chicago, IL, USA).

## 3. Results

The average duration of treatment was 6.3 (±4.3) days. On average, four treatment sessions (±2.0) were performed until the therapy was terminated. One patient quit the study at his own request.

### 3.1. Pain

**Figure 1:**
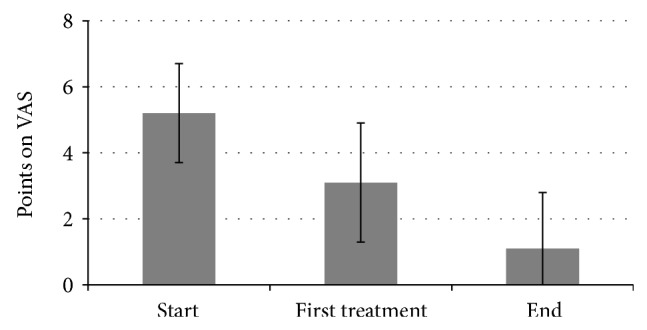
Score of pain in visual analogue scale.

During the course of the therapy, the average level of exercise-induced pain on the visual analogue scale (VAS) could be reduced from 5.2 ± 1.5 points to 1.1 ± 1.7 points (end of treatment) (*P* < 0.001; Figure 1). After the first treatment, the average level of pain sensation was already reduced to 3.1 ± 1.8 points (*P* < 0.001; Figure 1). Three patients were already pain-free after the first treatment (from 5 points to 0 points on the VAS). In total, 53% of patients were pain-free at the end of treatment (VAS: 0). Among the other 47%, the average level of complaints could be reduced from 5.3 ± 1 points to 2.3 ± 1.8 points on the VAS.

### 3.2. Pain-Free Running Distance

**Figure 2:**
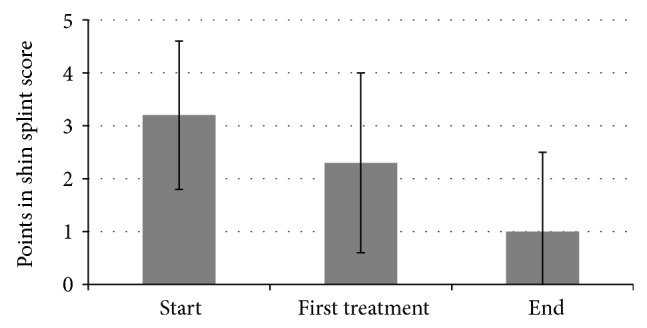
Running distance in shin splint score.

After completion of treatment, 60% of all participants could run a distance of more than 3000 m without pain. An improvement of the initial condition was achieved in further 19% of all participants. In these cases, the level of complaints was reduced from a score of 4.2 ± 0.4 to a score of 1.7 ± 0.8 (see Table 1). 19% reported that no improvement was observed. One patient reported a deterioration in his condition after the first treatment and quit the study at his own request. In total, the score could be improved from 3.2 to 1 (*P* < 0.001; Figure 2). 22% reported that they had run more than 3000 m (score reduced from 2.4 to 0) already after the first treatment. In total, the score for the running distance could be improved from 3.2 to 2.3 after the first treatment (*P* = 0.002; Figure 2).

### 3.3. Speed without Pain

**Figure 3:**
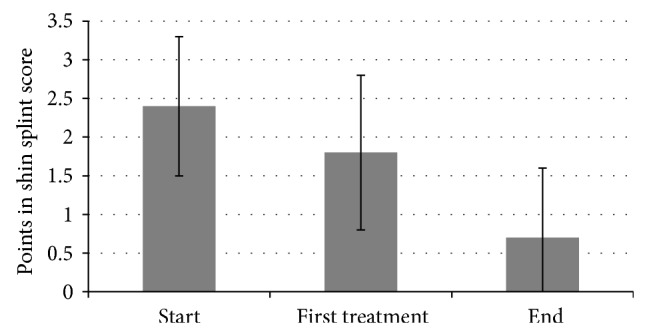
Velocity in shin splint score.

When the therapy was terminated, 56% of all patients managed to accomplish each of the speed levels specified in Table 1 without experiencing any pain. An improvement was achieved in 35% of all patients. Their score was improved from 2.7 to 1.4. In total, the score was improved from 2.4 to 0.7 (*P* < 0.001, Figure 3). In two cases, no effect could be achieved in terms of speed. One patient reported a deterioration in the score (score increased from 2 to 3). After the first treatment, 13% reported that they could run at each speed specified (score reduced from 2.3 to 0). In total, the score could be improved from 2.4 to 1.8 after the first treatment (*P* = 0.001; Figure 3).

### 3.4. Ability to Jump without Pain

**Figure 4:**
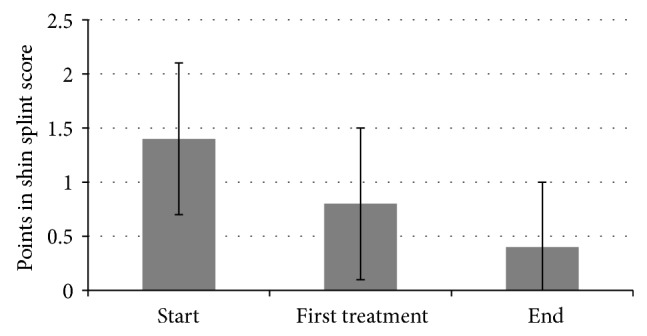
Jumping in shin splint score.

In total, the score for the ability to jump could be improved from 1.4 to 0.4 (*P* < 0.001; Figure 4). 66% of all patients stated that they were free of symptoms when the therapy was terminated (score reduced from 1.3 to 0). The ability to jump could be improved in further 22% of all patients (score reduced from 1.7 to 1). The therapy had no effect on the ability to jump in 9% of all cases. One patient experienced a deterioration (score increased from 1 to 1.5). 32% of all patients reported that they were able to jump without pain after the first treatment (score reduced from 1 to 0). In total, the score could already be improved from 1.4 to 0.8 after the first treatment (*P* < 0.001; Figure 4).

### 3.5. Shin Splint Score

**Figure 5:**
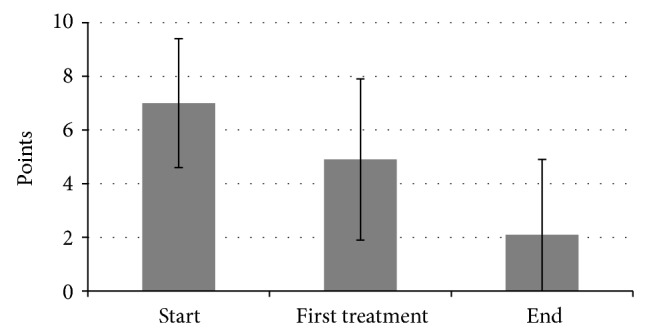
Shin splint score in total.

All capabilities sum to an exercise tolerance score with a maximum of 12 points. On average, the overall score was reduced from 7 (SD ± 2.4) to 2.1 (SD ± 2.8) (*P* < 0.001; Figure 5). 50% of all patients were completely free of symptoms after completion of treatment. Further 38% of all patients experienced an improvement in their symptoms (score reduced from 8 to 3.5). In 6% of all cases, it was not possible to prove an effect until termination of treatment. Two patients experienced a deterioration of their symptoms. After the first day of treatment, 13% of all patients were completely free of symptoms (score reduced from 6.8 to 0), and the overall score was improved from 7 to 4.9 after the first treatment (*P* < 0.001; Figure 5).

## 4. Discussion

The results of this clinical study show that the treatment method according to the fascial distortion model is a quick and effective option to relieve patients of pain and restore their exercise tolerance. In comparison Andrish et al. randomised 97 military recruits with MTSS into 5 groups to determine the effects of rest and ice, aspirin, phenylbutazone, calf-stretching, and plaster casting [25]. Recovery was based on the ability to run in comfort 500 meters or when no tenderness remained. No significant difference was noted between groups. Shock-absorbing insoles have a small significant effect [26]. The stated duration of treatment according to FDM is significantly shorter than the therapy periods indicated for shock wave therapy and physiotherapy [9, 16]. No therapeutic aids are required for the method applied, but it is time-consuming. In severe cases, a complete initial treatment can take up to one hour for each lower leg. The time expenditure will decrease in the subsequent treatment sessions. Moreover, the method is very painful for the patient, and the patient must be informed of this prior to treatment in order to improve the therapy tolerance. The immediate therapeutic success motivates the patient to complete the course of treatment. As described above, a significant improvement in pain and exercise tolerance is mostly achieved after the first treatment. In contrast, the treatment methods mentioned in the literature comprise treatment periods of up to 3 months [9]. With a treatment duration of about 7 days, the method according to the fascial distortion model presented is less time-consuming. However, the present study is limited by the fact that the patients were only available during a military sports course for a maximum period of three weeks. All patients who experienced an improvement but were not yet completely free of symptoms at the end of the course could not continue with their treatment until a completely asymptomatic condition would be reached, because the geographical distance to the treatment location was too far. This is the reason why in some cases the treatment had to be discontinued before an asymptomatic condition could be reached although an improvement was observed. Furthermore, this is a pilot study where long-term follow-up is not recorded. It has only been demonstrated that exercise tolerance can be restored within a short time by targeted manual intervention. An assessment of the therapeutic success in direct comparison with alternative treatment options was not possible. This will be necessary to prove that FDM is superior to other therapeutic approaches and should be part of further investigation.

However, the therapy according to the fascial distortion model proved to be superior to other physiotherapy methods in the management of shoulder pain syndromes [22, 23]. The functional approach of the therapy of MTSS is adequate, as it has not yet been possible to identify a tangible structural correlate [5]. Structural diseases should be excluded if there are relevant signs or if the functional therapy fails. These diseases include in particular compartment syndromes and stress fractures. Here fat suppressed magnet resonant imaging (MRI) is the method of choice [27, 28]. Results of MRI showed that 43.5% of the symptomatic legs showed bone marrow or periosteal edema. Absence of periosteal and bone marrow edema on MRI was associated with longer recovery [29]. Because of the small number of patients in our present pilot study associated with a missing control group and a short follow-up period with the patients the interpretation of the results is limited. Further comparative studies, including long-term observation and consideration of duration of the symptoms, should be conducted to demonstrate the superiority of the fascial distortion model to alternative therapies.

## Figures and Tables

**Table 1:** Scores for determining the exercise tolerance of patients.

Category	Points	Definition
Running, distance	5	Pain-free walking impossible
	4	Pain-free running impossible
	3	Pain-free running, approx. 400 m
	2	Pain-free running, approx. 1000 m
	1	Pain-free running, approx. 3000 m
	0	Pain-free running, more than 3000 m

Running, speed	4	Pain-free walking impossible
	3	Pain-free walking (approx. 3–5 km/h)
	2	Fast pain-free walking (approx. 6 km/h)
	1	Pain-free long distance running speed
	0	Pain-free sprint speed

Jumping	3	Jumping impossible
	2	Severe pain when jumping
	1	Slight pain when jumping
	0	No pain when jumping
